# Smart milking, smarter cows: A pilot study exploring voluntary dry-off in automatic milking systems

**DOI:** 10.3168/jdsc.2026-1093

**Published:** 2026-10-05

**Authors:** Megan Woodrum Setser, Elizabeth McGuire, Ryan Pralle, Jimena Laporta, Jennifer Van Os, Katherine Creutzinger

**Affiliations:** 1 Department of Animal Science, University of Connecticut, Storrs, CT 06269; 2 Department of Animal and Veterinary Sciences, University of Vermont, Burlington, VT 05405; 3 Department of Animal and Dairy Sciences, University of Wisconsin–Madison, Madison, WI 53706; 4 Animal Science, University of Wisconsin–Platteville, Platteville, WI 53818; 5 Ballchem Corporation, Montvale, NJ 07645

## Abstract

Automatic milking systems (AMS) can provide dairy cattle with agency over milking frequency, including when they transition from lactation to the dry period. The aim of this observational pilot study was to determine whether dairy cows would dry off voluntarily and to characterize self-milking behavior in late lactation. Holstein dairy cows (n = 15; 12 multiparous, 3 primiparous) were enrolled in a voluntary dry-off procedure in late lactation (mean ± SD: 74 ± 3 d days before expected calving) in which they no longer received concentrate in the AMS and were not fetched for milking. Cows were considered dry if they did not visit the AMS for 72 h or produced <4.5 kg milk/d for 3 d consecutively. If cows did not dry off by 40 d before expected calving, they were dried off by farm intervention. Twelve cows met the voluntary dry-off criteria 20.3 ± 8.0 d after enrollment (range: 10–32 d). From enrollment to the last day of milking, daily milk yield was reduced by 46.6% ± 18.9%, and the time between AMS visits increased from 9.6 ± 1.7 h to 25.1 ± 5.3 h. Daily milk yield had a high negative correlation with the interval between milkings, with higher daily milk yield corresponding to shorter intervals between visits. Additionally, cows with a greater percent reduction in milk yield by 9 d after enrollment and cows with more variable daily milk yield met the dry-off criteria earlier. This pilot study suggests that cows can reduce milking voluntarily, which may improve their agency and welfare. However, if cows are allowed to reduce milkings voluntarily, they still require monitoring during late lactation to ensure proper progression into the dry period.

## Graphical Abstract

**Figure:**
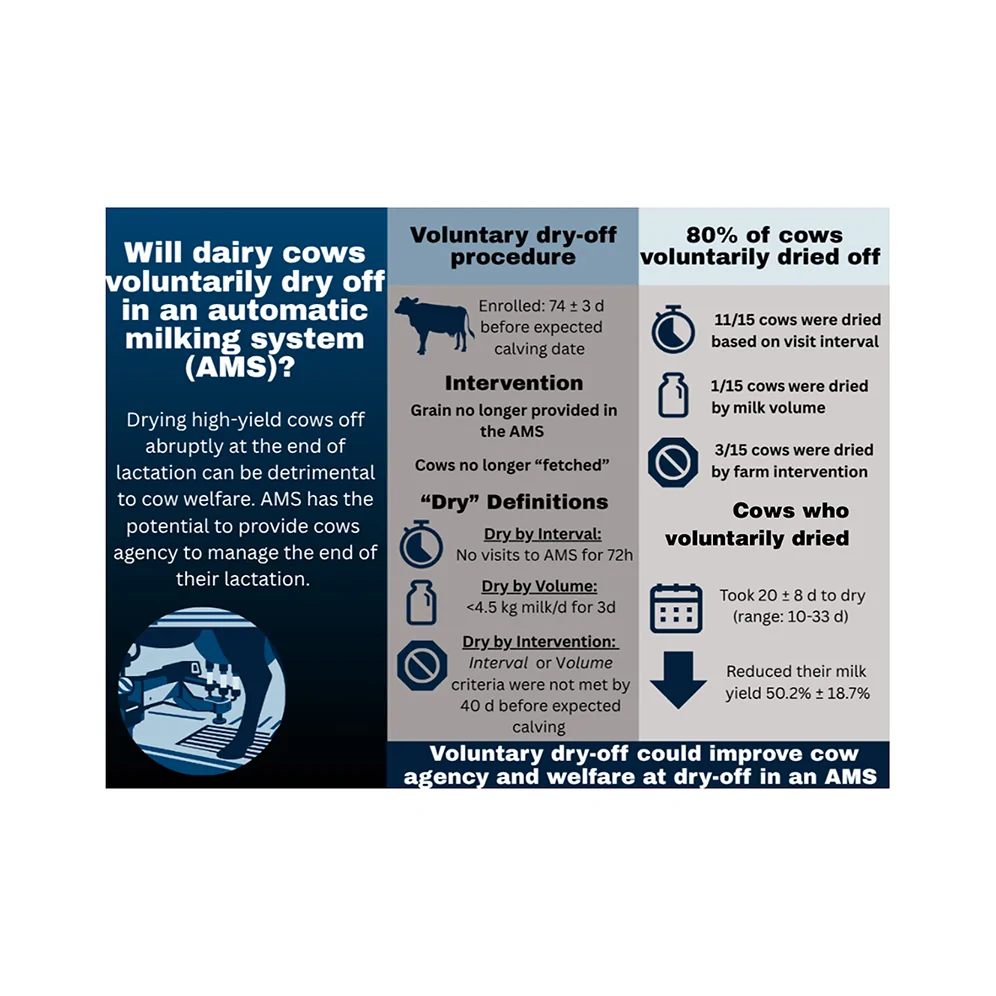

Cessation of milking, commonly called dry-off, marks the end of the lactation period for dairy cows. A non-lactation period (i.e., dry period) is important for mammary gland involution and epithelial regeneration to optimize milk yield in the subsequent lactation (Capuco et al., 1997; Van Knegsel et al., 2014). Most dairy producers dry cows off abruptly, immediately ceasing milking without a decrease in milking frequency before dry-off (73.6% of farms; USDA, 2016), to minimize labor needs compared with gradually reducing milking frequency for a specific set of late-lactation cows (Pattamanont et al., 2020).

Dairy cows frequently produce large volumes of milk due to genetic selection and optimized nutrition management (Barkema et al., 2015). Milk yield at the end of lactation and dry-off method both have important welfare and physiological effects for cows: cows who produced >21 kg on the last day of lactation compared with <21 kg/d had 1.8 times lower odds of quarters closing after dry-off due to increased milk leakage preventing keratin plug formation (Dingwell et al., 2004). Moreover, cows who produced >15 L on the last day of lactation were 4.8 times more likely to have clinical mastitis within 60 d of calving than cows producing <10 L/d (McDougall et al., 2022). High-yield cows also have greater fecal glucocorticoids (Bertulat et al., 2013) and plasma cortisol concentrations (Odensten et al., 2007) than low-yield cows when abruptly dried, possibly due to pain from udder engorgement.

Some dairy producers use a gradual dry-off method, such as a reduction in milking frequency or a low-energy diet (reviewed by Vilar and Rajala-Schultz, 2020), to reduce milk yield before cessation of milking for some or all their cows (USDA, 2016). Previous research found that reducing milking frequency by first skipping single and then multiple milkings over a 5-d period resulted in fewer cows with milk accumulation in the udder and leakage after dry-off compared with abruptly dried-off cows (Zobel et al., 2013). Gradual dry-off can also result in fewer behavioral signs of discomfort (a negative affective state originating from physical or mental stimuli; Franchi et al., 2024), such as more time lying and less time waiting at the gate to be milked compared with abruptly dried cows (Zobel et al., 2013; Rajala-Schultz et al., 2018). Despite these benefits, producers primarily use abrupt methods, likely due to the increased labor and management required to identify and gradually dry-off groups or individual cows.

Automatic milking systems (**AMS**) may offer an alternative approach to dry-off that could improve dairy cow welfare through the transition between the lactation and dry periods. Farms with AMS can adjust milking management at the individual level without increasing the time spent making decisions about and managing a subset of cows at each milking (Jacobs and Siegford, 2012). Cows are motivated to visit an AMS by the provision of a highly palatable concentrate and intramammary pressure from milk accumulation in the udder (Rathore, 1982; Madsen et al., 2010). Free-flow AMS allow cows free choice to enter the milking robot (Kappel et al., 2026) and can provide them with opportunities for agency to meet their need to alleviate udder pressure through milk removal.

In animal welfare, choice can be defined as the ability for an animal to choose between options or to use or not use a resource (elective use; Englund and Cronin, 2023). This ability to elect to use a resource allows animals a degree of control over the environment and autonomy that is essential to the psychological and affective states of animals (Leotti et al., 2010; Englund and Cronin, 2023). Should a farm cease to fetch cows (i.e., manually lead them to the AMS) and remove feed as a motivating factor to visit the AMS, then cows' motivation to use the AMS should be based on the desire to have milk removed from the udder. Elective use of an AMS may provide cows agency to self-regulate their transition from lactating to dry without requiring additional labor.

The objectives of this pilot observational study were to determine whether dairy cows with elective use of an AMS would meet voluntary dry-off criteria following the removal of the feed incentive from the milking robot, to characterize behavior around voluntary dry-off, and to determine whether milking characteristics were associated with the time for cows to dry themselves off. We hypothesized that cows would continue to visit the AMS after the feed incentive was removed, AMS visits would be driven by milk yield, and cows would have gradually increased AMS visit intervals and decreased milk yield, and would dry themselves off.

This study was performed at the University of Wisconsin–Platteville Pioneer Farm Dairy Center (Platteville, WI) from October 2023 to May 2025. Dairy cows in this study were housed in a freestall barn with 2 pens of 47 stalls and AMS robotic milker each (Lely Astronaut A5, Lely, Maassluis, the Netherlands; average rolling stocking density [mean ± SD]: pen 1/AMS 1 = 45 ± 2; pen 2/AMS 2 = 44 ± 2 cows). Cows stayed in the same pen (pen 1 = 12; pen 2 = 3) for the study duration. A partial mixed ration comprising corn silage haylage, corn, and a protein mineral mix formulated for lactating cow nutritional needs (NASEM, 2021) was provided ad libitum at the feed bunk, and feed was pushed up hourly to ensure continuous access to the diet. Before enrollment in the voluntary dry-off procedure, cows were provided with pelleted concentrate in the AMS during milking (Custom Mix, Cargill Animal Nutrition: 18% crude protein, 4.7% crude fat, 9% crude fiber). In the week before study enrollment, cows were fed an average of 3.6 ± 1.6 kg concentrate/d; the amount was determined by the Lely software (Lely Horizon) depending on individual cow daily milk yield.

Holstein dairy cows (n = 15; 12 multiparous [2.3 ± 0.7 lactations], 3 primiparous) were enrolled in the study weekly at 74 ± 3 d before their expected calving date. On the day of enrollment (d 0), cows were 304 ± 36 DIM and produced 33.1 ± 6.1 kg milk/d. This is the first study to explore elective use of an AMS for dry-off. As such, a sample size calculation was not performed, and the sample size was selected based on a previous study that explored voluntary use of a precision technology (Grinter et al., 2023). Cows were eligible for inclusion if they had SCC <300,000 cells/mL (obtained using Dairy Herd Improvement testing records), were not lame (locomotion score <3; 5-point scale; Sprecher et al., 1997), had a 10-d average of ≥2.2 successful AMS visits per day, and were not considered a chronic fetch cow by farm staff (regularly require staff to guide to the AMS). After enrollment, cows were no longer fetched for milking by farm staff and no longer provided concentrate in the AMS (voluntary dry-off procedure). Cows were managed as dry (i.e., no longer milked) when they did not visit the AMS for 72 consecutive hours (interval) or if they produced ≤4.5 kg of milk for 3 d consecutively (volume; <10% daily milk yield of 280 DIM herd average). If cows did not dry themselves off by 40 d before their expected calving date, they were dried off by farm staff (intervention) to ensure a 40-d dry period (Bachman and Schairer, 2003). At this point, cows were moved to a separate dry-cow facility, and no longer followed as part of this study.

The AMS automatically summarized milk yield per visit (kg), interval between visits (regardless of visit type; h), and visit type: successful visit (cow milked out), refusal (visited within 3.6 h following a successful visit and was not milked), and failures (cow was eligible and the machine failed to milk her out). The summed total of milk yield, number of successful visits, number of refusals, and average for the visit interval were recorded for each cow each day; failures were not evaluated, as this type of visit had 2 total occurrences. Data were extracted using the AMS software. From these data, milking characteristics were extracted for each cow, including the milk yield at enrollment (kg/d), time to dry-off (d), dry-off method (time, volume, intervention), percent change in milk yield, and daily magnitude change in milk yield. The percent change in milk yield was calculated as

$$\text{\textbackslash{}[}\frac{\left[Milk\;yield\;\left(kg\right)Day0\right]-\left[Milk\;yield\;\left(kg\right)Day_{i}\right]}{\left[Milk\;yield\left(kg\right)Day0\;\right]}\times 100,\text{\textbackslash{}]}$$

where Day0 is the day cows were enrolled and Day*_i_* is the day relative to enrollment. Daily magnitude change in milk yield was calculated as

$$\left|\left[Milk\;yield\;\left(kg\right)\;Day_{i}\right]-\;\left[Milk\;yield\;\left(kg\right)\;Day_{\left(i+1\right)}\right]\right|.$$

The average was then taken of this daily magnitude change in milk production (**AVMag**) by cow to represent typical magnitude change in a cow's milk yield, and the SD of the calculated daily magnitude change in milk yield (**SDMag**) quantified the variability in day-to-day magnitude changes for each cow. Together, AVMag and SDMag represent how consistent milk yield was for cows between days. The percent change in milk yield, AVMag, and SDMAG were calculated for key periods of interest. Data were analyzed using SAS (9.4, SAS Institute Inc., Cary, NC) and R Studio (R version 4.5.0, https://www.r-project.org/) based on specific analysis needs and tools provided by each software. Significance for all analyses were declared at *P* < 0.05.

The AMS visit interval, number of successful visits, and refusals were analyzed to determine whether cows altered their behavior from d 0 to the last day each cow milked before dry-off. Data from d 0 and the last day milked were screened for normality for selection of appropriate statistical tests through data visualization and normality tests (PROC UNIVARIATE; Shapiro–Wilk test). The AMS visit interval was normal for d 0 and the last day milked; a paired *t*-test (PROC TTEST) was used to compare AMS visit interval between these days. The number of successful visits and refusals on d 0 and the last day milked were not normally distributed; a Wilcoxon signed-rank test (PROC UNIVARIATE) was used to assess the difference in visit frequency between these days.

A repeated-measures correlation was performed to assess the within-cow relationship between daily milk yield (kg/d) and the average daily AMS visit interval (h; R studio, rmcorr; Bakdash and Marusich, 2017) by estimating a common regression slope across cows while accounting for the nonindependence of the repeated measures by day. The number of observations per cow was the number of days for her to meet the dry-off criteria. The strength of the correlation was interpreted according to Hinkle et al. (2003): <0.30 = negligible; 0.30 to <0.50 = low; 0.50 to <0.70 = moderate; 0.70 to <0.90 = high; and >0.90 = very high.

Daily milk yield, milk yield by visit, and interval between AMS visits data were visually assessed (Figure 1). Daily milk yield appeared to be nonlinear. Thus, a breakpoint analysis was performed to determine if there was a point between d 0 and dry-off at which the reduction in milk yield changed (following Creutzinger et al., 2021). Breakpoint analysis (PROC NLIN) was performed on pooled data across all cows and confirmed a break in the slope (change in milk yield by day) 9 d after enrollment. Thus, the pre-break and post-break periods were considered d 0 to 9 and d 10 to the last day of milking, respectively. An ANOVA with post hoc analysis (PROC MIXED) and Tukey adjustment was conducted to determine whether daily milk yield differed significantly between d 0, the last day of the pre-break period, and the last day cows were milked. Cows were included as a random variable and day treated as a repeated measure. If cows did not milk on the last day of the pre-break period, the last day cows were milked in this period was used (d 9 = 10, d 8 = 2, d 7 = 3 cows). For the milk yield on the last day each cow was milked, 2 cows were last milked during the pre-break period; their last day of milking values were excluded from the last-day-milked data to prevent repeated values in the model.

**Figure 1:**
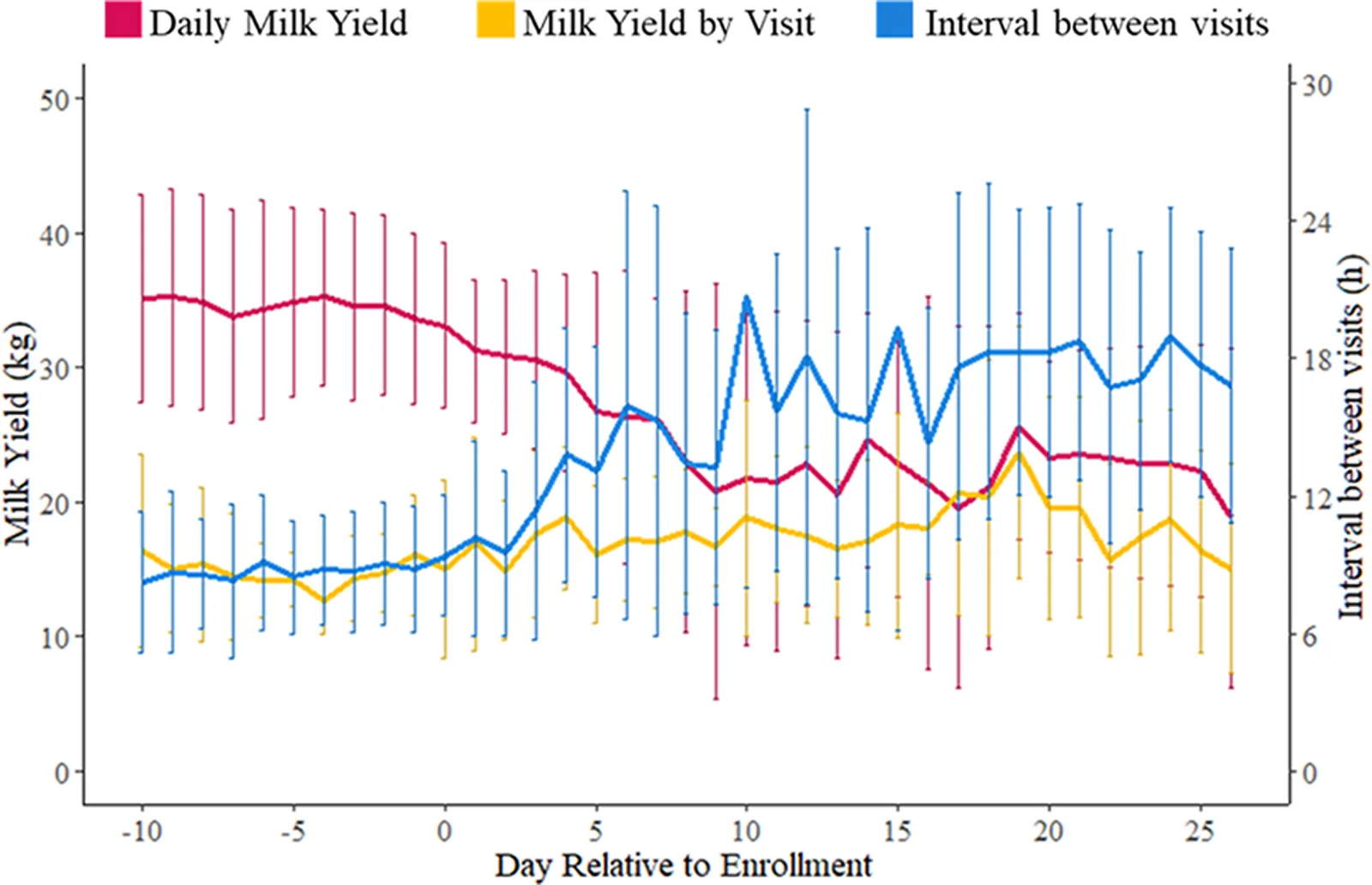
Descriptive data of the sample population, including the mean and SD (error bars) of the milk yield (kg), intervals between visits to the robotic milker (h), and average milk yield per visit (kg/visit) by day relative to enrollment in the voluntary dry-off procedure (d 0; n = 15 Holstein dairy cows). The left y-axis shows milk yield (kg) for the daily milk yield (magenta line) and the milk yield by visit (yellow line). The right y-axis shows hours for the interval between visits to the AMS (blue line).

The milking characteristics, milk yield at enrollment, percent change in milk yield, AVMag, and SDMag were calculated for the pre-break and post-break periods. For percent change in milk yield for the pre-break period, if a cow did not visit the AMS on d 9, the last day the cow was milked before the end of the pre-break period, defined previously, was used in the calculation. Two cows met voluntary dry-off criteria and were not included in the post-break period values due to lack of milking events.

Additional analysis was conducted to determine whether early milking characteristics following enrollment were associated with the number of days to dry-off. Separate linear regressions were used to determine whether milking characteristics in the pre-break period (milk yield at enrollment, total percent change from d 0 to 9 relative to enrollment, AVMag, and SDMag) were associated with the number of days from enrollment until dry-off. Univariable analysis was used to determine whether parity (primiparous vs. multiparous), season enrolled (winter: December–February, spring: March–May, summer: June–August, fall: September–November), DIM at enrollment, and pen (1 vs. 2) would be included as independent variables in the multivariable model (included if *P* ≤ 0.20). None of these variables met our inclusion criteria. Residuals were visually assessed with quantile–quantile and histogram plots for all models to evaluate model assumptions.

Of the 15 cows enrolled, 12 met the voluntary dry-off criteria before 40 d prior to expected calving (80%): 11 cows did not visit for 72 h, and 1 cow had milk yield <4.5 kg/d for 3 consecutive d. Cows who met voluntary dry-off criteria reached the dry-off criteria (mean ± SD) 20.3 ± 8.0 d (range: 10–32 d) after enrollment. For cows dried off according to the interval definition, the last day milked was 72 h (3 d) before the dry-off date, 17 ± 8 d after enrollment. Cows who were dried off by farm intervention were milked for 37 ± 4 d after enrollment. Daily milk yield was reduced by 46.6% ± 18.9% for all cows over the study period (d 0 = 33.1 ± 6.1 kg/d, vs. last day milked = 17.5 ± 6.7 kg/d). When broken down by dry-off method, the total percent reduction in daily milk yield was 50.2% ± 18.7% (d 0 = 32.7 ± 6.6 kg/d, vs. last day milked = 16.1 ± 6.7 kg/d) for cows who met voluntary dry-off criteria (n = 12), and 32.2% ± 13.7% (d 0 = 34.7 ± 17.6 kg/d, vs. last day milked = 23.2 ± 11.8 kg/d) for cows dried by intervention (n = 3).

Milking behavior was altered for all cows after enrollment. Daily milking frequency was reduced from d 0 to the last day of milking (median [first to third quartile range]: 2.0 [2.0–3.0] vs 1.0 [1.0–1.0] milkings, respectively; S = 55.5; *P* < 0.001). On d 0 and the last day each cow was milked, the AMS visit interval was 9.6 ± 1.7 h (mean ± SD) and 25.1 ± 5.3 h (voluntary = 25.7 ± 5.1 h, vs. intervention = 19.5 ± 7.3 h), respectively. Cows increased AMS visit interval from d 0 to the last day each individual cow was milked by (mean difference ± SE) 15.5 ± 1.4 h [95% CI: 12.6–18.4 h] regardless of dry-off method (T = −11.5; *P* < 0.001). Additionally, daily milk yield was highly correlated with the average daily AMS visit intervals (r = −0.83 [95% CI: −0.86 to −0.80]; *P* < 0.001); cows had shorter intervals between AMS visits when daily milk yield was higher.

Overall, daily milk yield was reduced following enrollment in the voluntary dry-off procedure. A nonlinear relationship in the change in daily milk yield was identified. The change in milk yield by day was significantly different between d 0 to d 9, and from d 9 to the last day of milking (slope days ≤9 = −1.40 vs. slope days >9 = −0.18; *P* < 0.001). Milk yield was reduced by (mean ± SE) 7.8 ± 2.7 kg from d 0 to the last day cows were milked in the pre-break period (33.1 ± 1.9 [95% CI: 29.1–37.0] kg/d vs. 25.2 ± 2.0 [95% CI: 21.2–29.2] kg/d, respectively; adjusted *P* = 0.02; Figure 2). Milk yield was further reduced by 7.7 ± 2.7 kg from the end of the pre-break period to the last day each cow was milked (17.4 ± 2.1 [95% CI: 13.2–21.8] kg/d on the last day milked; adjusted *P* = 0.03). Thus, overall milk yield was reduced 15.6 ± 2.9 kg from d 0 to the last day each cow was milked (adjusted *P* < 0.001).

**Figure 2:**
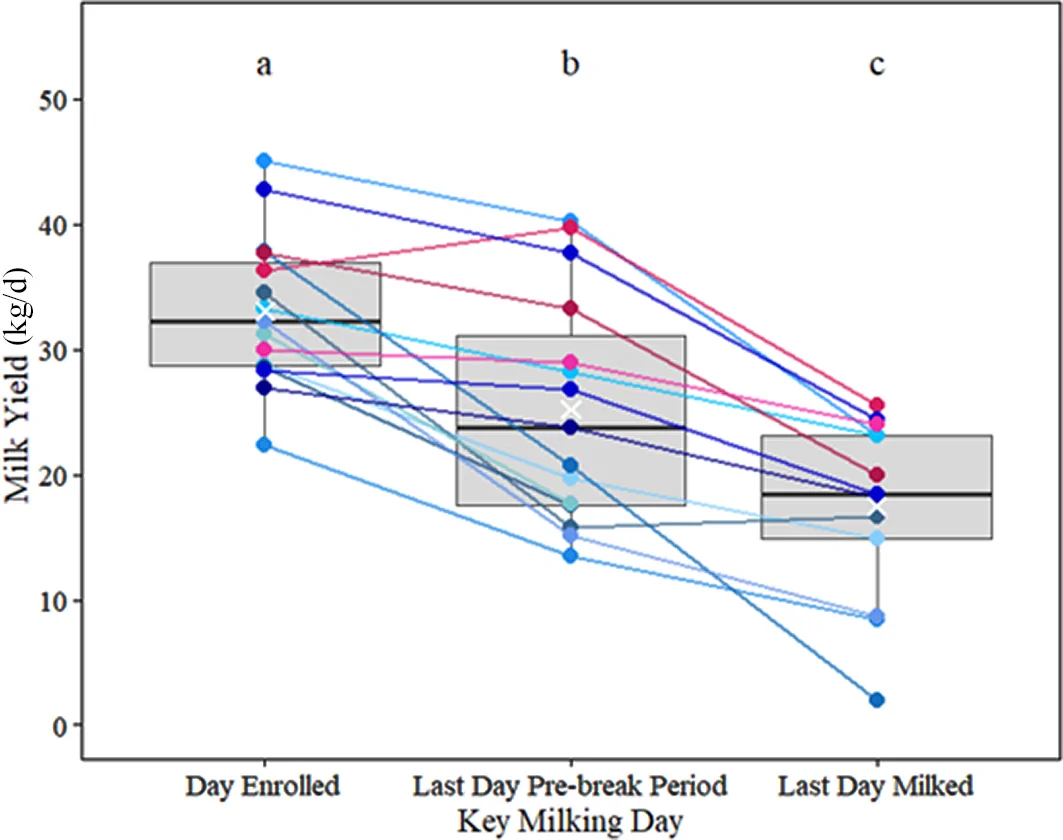
Box plots showing the median (line) and interquartile range (box) of the milk yield (kg) on the day enrolled in the voluntary dry-off procedure (n = 15), the last day milked at the end of the pre-break period (day 0–9 following enrollment; Last Day Pre-break Period, n = 15), and the last day cows were milked prior to dry-off (n = 13 Holstein dairy cows). Two cows were not included in the measures for the last day cows were milked because their last day milked was in the pre-break period. White X's indicate mean milk yield for cows on each key milking day. Each dot represents an individual cow, and the color for each individual stays consistent across days. Dots in shades of blue represent cows who met voluntary dry-off criteria; dots in shades of pink and red represent cows who were dried by farm intervention. The lines connecting points track the change in milk yield by each individual enrolled between the key milking days. Significant differences in daily milk yield between key milking days are denoted by different letters (*P* < 0.05).

Cows showed considerable variation in their daily average magnitude. The AVMag was (mean ± SD) 3.7 ± 1.6 Δ kg/d by cow (range: 1.6 to 6.1 average Δ kg/d by cow) during the entire enrollment period, 2.7 ± 1.9 Δ kg/d (0.9 to 6.5 Δ kg/d by cow) in the pre-break period, and 5.3 ± 4.6 Δ kg/d (1.2 to 18.6 Δ kg/d by cow) in the post-break period. The SDMag was 5.4 ± 2.1 Δ kg/d (1.3 to 9.5 SD Δ kg/d by cow) over the entire enrollment period, 3.1 ± 2.7 Δ kg/d (0.6 to 8.3 Δ kg/d by cow) in the pre-break period, and 5.9 ± 2.6 Δ kg/d (0.7 to 10.5 Δ kg/d by cow) in the post-break period.

Milking characteristics in the pre-break period were associated with the time it took cows to dry off. Although daily milk yield on d 0 was not associated with the time to dry-off (*F* = 2.6; *P* = 0.13), cows with a greater percent change in milk yield over the pre-break period were dried off sooner than cows with a smaller change in milk yield (intercept = 31.3 [95% CI: 24.3–38.4], slope −0.3 [95% CI: −0.5 to −0.1]; *F* = 9.3; *P* = 0.009). Additionally, cows with greater AVMag (intercept = 33.3 [95% CI: 25.6–41.0], slope = −3.6 [95% CI: −6.0 to −1.3]; *F* = 11.0; *P* = 0.006) and cows with a greater SDMag (intercept = 31.4 [95% CI: 25.1–37.7], slope = −2.6 [95% CI: −4.1 to −1.0]; *F* = 12.7; *P* = 0.004) dried off sooner than cows with smaller AVMag and SDMag, respectively.

In this exploratory study, it took between 10 and 32 d for cows to dry off if they met voluntary dry-off criteria (12/15 cows) before 40 d prior to expected calving. Providing dairy cows with elective AMS use without a feed incentive allows them the opportunity to self-regulate their milk removal needs at an individual level without an external motivator (Englund and Cronin, 2023). In this case, providing cows the agency to meet their motivation to be milked and to respond accordingly to a reduced milk yield may allow an individualized transition into the dry period and prevent negative affective states such as discomfort (Mellor, 2015; Špinka, 2019). However, we did not directly measure motivation and affective states after implementing the voluntary dry-off procedure, and future research could more explicitly investigate these aspects.

The observed decrease in the number of visits, the increased time between AMS visits, and the inverse relationship between milk yield and time between AMS visits support the connection between milk yield and motivation to be milked. Sensitivity to and amount of intramammary pressure from milk accumulation in the udder likely drive the observed relationship between AMS visit interval and milk yield (Witzel and McDonald, 1965; Ouweltjes, 1998). The point at which cows stopped visiting the AMS may reflect the point at which the motivation to be milked no longer outweighed the work to visit the AMS, particularly with its reduced reward value with the concentrate incentive removed. The observed relationship between milk yield and visit interval are consistent with previous research that also found a negative correlation between milk yield and milking interval at the individual level throughout the lactation for cows milked in an AMS, although in that study cows still received a concentrate incentive in the AMS and were fetched by farm staff if they did not visit the AMS for 20 h (Løvendahl and Chagunda, 2011). The withdrawal of concentrate and resulting decline in energy supply could have led to reduced milk synthesis and accumulation in the udder, contributing to a lower motivation to visit the milking unit. Comparatively, France et al. (2022) found that cows fed restricted feed in an AMS before dry-off (wk −2 = 0.75 kg/d, wk −1 = 0.5 kg/d) visited the AMS 2.4 times/d compared with 2.7 times/d for cows fed unrestricted concentrate, demonstrating that cows continued to visit the AMS even with reduced dietary energy from AMS concentrate. Though our study did not provide concentrate in the AMS, we suspect that the change in provision of dietary energy had minimal influence on daily AMS visits. This observed correlation between milk yield and visit interval at the individual level highlights the importance of considering individual cows at dry-off.

Of the cows who met voluntary dry-off criteria, a majority did not visit the AMS for 72 consecutive hours, likely indicating that cows were satisfied to cease milking above the 4.5 kg/d milk yield threshold set as the other dry-off criteria. The average milk yield of the voluntarily-dried cows on the last day they were milked was ∼16 kg/d, a 50% average reduction in milk yield from d 0. Milk yield at dry-off for the voluntarily dried cows was less than the milk yield observed in Holstein cows milked in an AMS subjected to a restricted feed or restricted milking schedule for 2 wk before dry-off, who produced between 19 and 22 kg milk/d on the last day of milking (France et al., 2022). Additionally, the percent reduction in milk yield before dry-off for cows who dried voluntarily was greater than the 33% reduction in milk yield observed in dairy cows across 5 dairy farms who were gradually dried off at >300 DIM by reducing to a once-daily milking for the last week of their lactation (reduced from 19.8 ± 0.4 kg/d to 13.2 ± 0.4 kg/d; Gott et al., 2016). These results indicate that cows in the present study had a greater reduction in milk yield when given the opportunity to self-select the last day of milking than was observed in previous gradual dry-off studies, though these comparisons are limited due to varying conditions under which these studies were conducted. Therefore, the traditional approaches to reducing milking frequency or feed to reduce milk yield for set times before dry-off may not allow cows to adequately meet their motivations to remove milk from their udder. Based on these results, we hypothesize that udder capacity is likely more important than daily milk weights for dry-off timing. This study may serve as a guide for an approach to gradually dry off cows according to percent change in milk yield, which would account for individual udder capacity. Future research should follow cows to assess for milk-leaking, keratin plug formation, and time for udder involution to occur in relationship to self-dry-off. This research should also follow cows into their subsequent lactation to determine whether dry-off method affects calving or production in the next lactation, and should incorporate producer perspectives on dry-off options to provide a holistic view of voluntary dry-off as an option for commercial farms with AMS.

There was a greater reduction in daily milk yield from d 0 to d 9 than from d 9 to the last day of milking (−1.4 kg/d vs. −0.18 kg/d). The rate of change from d 0 to 9 d following enrollment is similar to the change in daily milk yield from studies implementing gradual dry-off procedures. Three studies implementing gradual dry-off procedures saw rates of change in the average milk yield of −2.6 kg/d (Zobel et al., 2013), −1.1 kg/d (Odensten et al., 2007), and −1.4 kg/d (Rajala-Schultz et al., 2018). Therefore, the voluntary dry-off procedure produced effects on the rate of change in milk yield of cows similar to those of a gradual dry-off procedure. However, 100% of the cows in our study continued to visit the AMS for longer than the gradual dry-off period of any of the aforementioned studies, perhaps indicating that cows may benefit from more time to transition from lactating to the dry period than the 5 or 7 d provided in some gradual dry-off procedures. However, we hypothesize that the marked reduction in the rate of decline in milk yield following d 9 may support that manually drying off cows after 9 d of a voluntary dry-off or gradual dry-off procedure could be sufficient to offset the aversive effects of abrupt dry-off. Future research could verify whether these observed changes in the rate of decline in milk yield apply beyond our study population.

Finally, this pilot study aimed to determine whether milking characteristics were associated with how long cows took to dry off following enrollment in the voluntary dry-off procedure. Milk yield at enrollment was not associated with how long it took cows to dry off. However, cows with a greater percentage change in milk yield in the pre-break period were dried off sooner. In addition to the previously described outcomes, cows who had greater AVMag and were more variable in their daily magnitude change in milk yield (greater SDMag) took fewer days to dry off. Previous evaluation of consistency versus variability in milk yield in dairy cows has been largely focused on the cow's ability to maintain milk yield or recover milk yield more quickly in the face of a disease challenge (i.e., resilience; Colditz and Hine, 2016; Colditz, 2022). However, in the context of this study, the greater initial relative change in milk yield and greater variability may lend itself to ease of adaptation into the dry period. Thus, context is essential to consider when interpreting whether consistency or variability may be more beneficial to dairy cows in late lactation. It is also possible that individual differences in behavior, driven by animal personality, played a role in the observed transition into the dry period. Animal personalities have been linked to behavioral flexibility, responsiveness to environmental stimuli, and ultimately adaptability (Wolf et al., 2008), and have been previously linked with dairy cow adaptability to AMS at the start of lactation (Brasier et al., 2023). We hypothesize that the variability between cows observed following enrollment in the voluntary dry-off procedure may suggest that personality, responsiveness, and behavioral flexibility could play a role in the transition into the dry period. However, further research is needed to better understand how individual differences are associated with the adaptability of dairy cows at the end of their lactation. Ultimately, milking characteristics in the first 9 d following enrollment of our study were associated with the time cows took to dry off, which may provide additional insight into individual cow needs at dry-off.

It is important to acknowledge that this research was conducted with a relatively small sample size on a single farm and was designed to characterize individual elective use of precision technologies. Thus, caution should be used when generalizing results, and future research should be performed with additional animals to improve our understanding of cow motivations to be milked at the end of lactation. A limitation of this study was the inability to isolate the effect of loss of feed from the AMS on cow behavior and change in milk yield. For instance, the loss of concentrate in the AMS may also have contributed to the reduction in milk yield observed in our study. Tucker et al. (2009) compared change in milk yield between abruptly and gradually dried cows who received either 8 or 16 kg/d DM; cows who received 8 kg/d DM in the gradual dry-off treatment saw the greatest reduction in milk yield (Tucker et al., 2009). However, cows in our study had unrestricted access to the partial mixed ration at the feed bunk and may have had compensatory intake for the loss of concentrate, but we were unable to measure feed intake at the feed bunk in this study. It is important to note that this study was also unable to account for the effects of feed motivation on persistence to visit the AMS. Highly food-motivated cows may have visited the AMS regardless of milk yield, in hopes of receiving a feed reward (Prescott et al., 1998). Future research should incorporate measures of feed motivation and total DMI, including at the feed bunk, to better understand the role of removing feed on voluntary dry-off, and should incorporate control treatments.

## References

[bib1] Bachman K.C., Schairer M.L. (2003). Invited review: Bovine studies on optimal lengths of dry periods. J. Dairy Sci..

[bib2] Bakdash J.Z., Marusich L.R. (2017). Repeated measures correlation. Front. Psychol..

[bib3] Barkema H.W., von Keyserlingk M.A.G., Kastelic J.P., Lam T.J.G.M., Luby C., Roy J.-P., LeBlanc S.J., Keefe G.P., Kelton D.F. (2015). Invited review: Changes in the dairy industry affecting dairy cattle health and welfare. J. Dairy Sci..

[bib4] Bertulat S., Fischer-Tenhagen C., Suthar V., Möstl E., Isaka N., Heuwieser W. (2013). Measurement of fecal glucocorticoid metabolites and evaluation of udder characteristics to estimate stress after sudden dry-off in dairy cows with different milk yields. J. Dairy Sci..

[bib5] Brasier J.E., Schwanke A.J., DeVries T.J. (2023). Effects of dairy cows' personality traits on their adaptation to an automated milking system following parturition. J. Dairy Sci..

[bib6] Capuco A.V., Akers R.M., Smith J.J. (1997). Mammary growth in Holstein cows during the dry period: Quantification of nucleic acids and histology. J. Dairy Sci..

[bib7] Colditz I.G. (2022). Competence to thrive: resilience as an indicator of positive health and positive welfare in animals. Anim. Prod. Sci..

[bib8] Colditz I.G., Hine B.C. (2016). Resilience in farm animals: Biology, management, breeding and implications for animal welfare. Anim. Prod. Sci..

[bib9] Creutzinger K.C., Dann H.M., Krawczel P.D., Habing G.G., Proudfoot K.L. (2021). The effect of stocking density and a blind on the behavior of Holstein dairy cattle in group maternity pens. Part I: Calving location, locomotion, and separation behavior. J. Dairy Sci..

[bib10] Dingwell R.T., Leslie K.E., Schukken Y.H., Sargeant J.M., Timms L.L., Duffield T.F., Keefe G.P., Kelton D.F., Lissemore K.D., Conklin J. (2004). Association of cow and quarter-level factors at drying-off with new intramammary infections during the dry period. Prev. Vet. Med..

[bib11] Englund M.D., Cronin K.A. (2023). Choice, control, and animal welfare: definitions and essential inquiries to advance animal welfare science. Front. Vet. Sci..

[bib12] France A.E., Dufour S., Kelton D.F., Barkema H.W., Kurban D., DeVries T.J. (2022). Effect of dry-off management on milking behavior, milk yield, and somatic cell count of dairy cows milked in automated milking systems. J. Dairy Sci..

[bib13] Franchi G.A., Bagaria M., Boswijk H., Fàbrega E., Herskin M.S., Westin R. (2024). Animal discomfort: A concept analysis using the domesticated pig (*Sus scrofa*) as a model. Livest. Sci..

[bib14] Gott P.N., Rajala-Schultz P.J., Schuenemann G.M., Proudfoot K.L., Hogan J.S. (2016). Intramammary infections and milk leakage following gradual or abrupt cessation of milking. J. Dairy Sci..

[bib15] Grinter L.N., Mazon G., Costa J.H.C. (2023). Voluntary heat stress abatement system for dairy cows: Does it mitigate the effects of heat stress on physiology and behavior?. J. Dairy Sci..

[bib16] Hinkle D.E., Wiersma W., Jurs S.G. (2003).

[bib17] Jacobs J.A., Siegford J.M. (2012). Invited review: The impact of automatic milking systems on dairy cow management, behavior, health, and welfare. J. Dairy Sci..

[bib18] Kappel S., Weary D.M., von Keyserlingk M.A.G. (2026). Invited review: Assessing dairy cow agency in automatic milking systems. J. Dairy Sci..

[bib19] Leotti L.A., Iyengar S.S., Ochsner K.N. (2010). Born to choose: The origins and value of the need for control. Trends Cogn. Sci..

[bib20] Løvendahl P., Chagunda M.G.G. (2011). Covariance among milking frequency, milk yield, and milk composition from automatically milked cows. J. Dairy Sci..

[bib21] Madsen J., Weisbjerg M.R., Hvelplund T. (2010). Concentrate composition for automatic milking systems—Effect on milking frequency. Livest. Sci..

[bib22] McDougall S., Williamson J., Gohary K., Lacy-Hulbert J. (2022). Risk factors for clinical or subclinical mastitis following infusion of internal teat sealant alone at the end of lactation in cows with low somatic cell counts. N. Z. Vet. J..

[bib23] Mellor D.J. (2015). Positive animal welfare states and encouraging environment-focused and animal-to-animal interactive behaviours. N. Z. Vet. J..

[bib24] NASEM (National Academies of Sciences, Engineering, and Medicine) (2021).

[bib25] Odensten M.O., Berglund B., Waller K.P., Holtenius K. (2007). Metabolism and udder health at dry-off in cows of different breeds and production levels. J. Dairy Sci..

[bib26] Ouweltjes W. (1998). The relationship between milk yield and milking interval in dairy cows. Livest. Prod. Sci..

[bib27] Pattamanont P., Marcondes M., De Vries A. (2020). Advantages and disadvantages of various dry-off methods for dairy cows. EDIS.

[bib28] Prescott N.B., Mottram T.T., Webster A.J.F. (1998). Relative motivations of dairy cows to be milked or fed in a Y-maze and an automatic milking system. Appl. Anim. Behav. Sci..

[bib29] Rajala-Schultz P.J., Gott P.N., Proudfoot K.L., Schuenemann G.M. (2018). Effect of milk cessation method at dry-off on behavioral activity of dairy cows. J. Dairy Sci..

[bib30] Rathore A.K. (1982). Order of cow entry at milking and its relationships with milk yield and consistency of the order. Appl. Anim. Ethol..

[bib31] Špinka M (2019). Animal agency, animal awareness and animal welfare. Anim. Welf..

[bib32] Sprecher D.J., Hostetler D.E., Kaneene J.B. (1997). A lameness scoring system that uses posture and gait to predict dairy cattle reproductive performance. Theriogenology.

[bib33] Tucker C.B., Lacy-Hulbert S.J., Webster J.R. (2009). Effect of milking frequency and feeding level before and after dry off on dairy cattle behavior and udder characteristics. J. Dairy Sci..

[bib34] USDA (2016).

[bib35] Van Knegsel A.T.M., Hammon H.M., Bernabucci U., Bertoni G., Bruckmaier R.M., Goselink R.M.A., Gross J.J., Kuhla B., Metges C.C., Parmentier H.K., Trevisi E., Tröscher A., Vuuren A.M.V. (2014). Metabolic adaptation during early lactation: Key to cow health, longevity and a sustainable dairy production chain. CABI Reviews.

[bib36] Vilar M.J., Rajala-Schultz P.J. (2020). Dry-off and dairy cow udder health and welfare: Effects of different milk cessation methods. Vet. J..

[bib37] Witzel D.A., McDonald J.S. (1965). Intramammary pressures between milkings in the lactating cow. J. Dairy Sci..

[bib38] Wolf M., van Doorn G.S., Weissing F.J. (2008). Evolutionary emergence of responsive and unresponsive personalities. Proc. Natl. Acad. Sci. USA.

[bib39] Zobel G., Leslie K., Weary D.M., von Keyserlingk M.A.G. (2013). Gradual cessation of milking reduces milk leakage and motivation to be milked in dairy cows at dry-off. J. Dairy Sci..

